# The global impact of wild pigs (*Sus scrofa*) on terrestrial biodiversity

**DOI:** 10.1038/s41598-021-92691-1

**Published:** 2021-06-24

**Authors:** Derek R. Risch, Jeremy Ringma, Melissa R. Price

**Affiliations:** 1 University of Hawai‘i at Mānoa, 1910 East-West Rd., Honolulu, HI 96822 USA; 2 Royal Melbourne Institute of Technology University, GPO Box 2476V, Melbourne, 3001 Australia

**Keywords:** Ecology, Systems biology, Ecology, Environmental sciences

## Abstract

The International Union for the Conservation of Nature’s (IUCN) Red List of Threatened Species is a comprehensive database of over 120,000 species and is a powerful tool to evaluate the threat of invasive species to global biodiversity. Several problematic species have gained global recognition due to comprehensive threat assessments quantifying the threat these species pose to biodiversity using large datasets like the IUCN Red List of Threatened Species. However, the global threat of wild pigs (*Sus scrofa*) to biodiversity is still poorly understood despite well-documented ecosystem level impacts. In this study, we utilized the IUCN Red List to quantify the impacts of this globally distributed species throughout its native and non-native range. Here we show that wild pigs threaten 672 taxa in 54 different countries across the globe. Most of these taxa are listed as critically endangered or endangered and 14 species have been driven to extinction as a direct result of impacts from wild pigs. Our results show that threats from wild pigs are pervasive across taxonomic groups and that island endemics and taxa throughout the non-native range of wild pigs are particularly vulnerable.

## Introduction

Global biodiversity is decreasing at an alarming rate with species extinction rates 1000 times greater than natural background rates and anticipated to be 10,000 times greater in the future^1^. The establishment and spread of invasive species are among the primary drivers of these losses as they directly affect native species and can influence ecosystem change through disturbance^2–4^. Understanding the processes by which invasive species affect native biota and the extent of their impact throughout their introduced range is a critical step in helping inform conservation actions to mitigate further losses in biodiversity. Threat assessments of widespread invasive species based on comprehensive databases like the International Union for the Conservation of Nature’s (IUCN) Red List of Threatened Species^5^ are commonly used to highlight regions and species that are most vulnerable, and to guide the prioritization of conservation actions. To date, a handful of invasive predators, such as rats, Indian mongoose, domestic dogs, and feral cats, have gained notoriety due to global assessments highlighting their distribution, generalist diet, and the extent of their impacts on species of concern^4,6–8^. However, the global impacts of wild pigs (*Sus scrofa*) are still poorly understood despite similarities in their global distribution and potential for multi-scale impacts on biodiversity and ecosystem services^9–11^.

Wild pigs are identified as one of the “100 of the World’s Worst Invasive Alien Species” alongside other more frequently discussed invasive terrestrial species such as cats (*Felis catus*) and rats (*Rattus rattus*)^12^. However, pigs are unique among other problematic terrestrial invasive species in that they are omnivorous generalists and function as both large predators and herbivores throughout their native and non-native range^13^. They have been documented predating upon a variety of vertebrate and invertebrate species in island and continental ecosystems^14–16^, disturbing nest sites and plant assemblages^17,18^, hybridizing with other endangered *Suidae*^19^, competing with native fauna^20,21^, and acting as vectors for disease transmission^13,22^. In addition to their direct impacts on both wildlife and plant communities, they are generally known to disturb ecosystem structure due to their unique rooting and digging behavior^23^. Consequentially, pigs are considered ecosystem engineers, having considerable secondary effects on organisms by physically altering habitat characteristics^13^. Despite these well-documented impacts from wild pigs on species and ecosystems, the degree to which wild pigs pose a threat to biodiversity at large is still largely unknown.

Although global summaries of pig impacts do exist, they have either been global qualitative papers drawing implications from many small-scale quantitative studies^9,10,13,24–27^ or large-scale quantitative studies addressing a specific process through which pigs threaten the environment (predation, herbivory, or ecosystem engineering)^4,11,28^ or their impacts on a particular ecosystem type^29^ or region^11^. These studies have undoubtedly led to a better understanding of the impact of wild pigs throughout their native and non-native range and have amplified interest in wild pig research in recent years, raising growing concerns among conservationists, agriculturalists, and the broader public that have been affected by wild pigs. These global qualitative review papers are helpful in identifying the processes through which pigs threaten ecosystems, but fall short in providing detailed quantitative information about the number, type, and location of vulnerable species. As a result, a comprehensive quantitative assessment addressing the potential role of pigs in global biodiversity declines does not currently exist.

Global assessments of threats to at-risk taxa have improved the effectiveness of conservation decision making by incorporating that understanding into the decision-making process^30^. By identifying regions and taxa most vulnerable to threats from invasive and pest species, conservation organizations have been able to address biodiversity loss in a more targeted manner. For example, the systematic eradication of introduced mammals on islands over the past few decades came as a direct response to an increasing body of literature implicating several introduced mammalian species largely responsible for island biodiversity declines^7,8,31^. Due to these coordinated and targeted efforts, rodents, goats, and cats have been the most commonly eradicated taxa on islands despite a similar distribution and potential for impact from wild pigs^32,33^. In addition to increasing the effectiveness of conservation interventions, a broader understanding of the threats to at-risk taxa from introduced or pest species highlight research deficiencies and allow for a more targeted allocation of research efforts on understudied or poorly understood systems and species^34^. To effectively mitigate impacts from wild pigs throughout their global range and allocate research efforts to understudied areas and taxa, we need a better understanding of their role in the declines of global biodiversity.

In this paper, we quantify the extent of threats from pigs to both plants and wildlife including all processes by which pigs threaten these taxa as described on the IUCN Red List and all terrestrial taxa with sufficient assessment information. Using this information, we enumerate how many taxa are threatened by wild pigs on the IUCN Red List and which taxonomic groups are most vulnerable. We also identify which types of impact are most prevalent and which regions globally can be considered critical in terms of impacts from pigs. Furthermore, we provide a comparative analysis of threats from pigs to both island endemics, continental taxa, and taxa threatened within the native and non-native ranges of wild pigs, and conclude by identifying pressing research and management priorities.

## Methods

We acquired a complete copy of the IUCN Red List of Threatened Species for all terrestrial taxa in May 2018^5^. For the purposes of this assessment, taxa refers to each unique listing on the IUCN Red List which is most frequently at the species level but also may include subspecies and variety. To exclude taxa that occur outside of the global range of wild pigs we acquired native range information for pigs from the IUCN Red List and non-native range information from Lewis et al. 2017^33^. Due to differences in resolution between range information provided for each taxon on the IUCN Red List and range information acquired for pigs, most range information was delineated at the country level. However, where more detailed and accurate range information was provided for both pigs and IUCN taxa, range information was delineated at the next available administrative boundary (e.g. states or provinces). Taxa were included initially that occurred in areas where wild pigs were described as potentially occurring by Lewis et al. 2017. However, if no taxa were found to be threatened by wild pigs within these ranges, all taxa in these areas were excluded. All other taxa with ranges exclusively outside of the native or non-native range of wild pigs were excluded from the final dataset. This large database was filtered using a systematic keyword search in R version 3.6.0 to identify keywords from the “Major Threats” section for each taxa that contained any of the following keywords: Pig, pig*, pigs, domesticus, Sus, scrofa, boar, boar*, boars, hog, hog*, hogs, swine^35^. This list of keywords was compiled based on commonly used names to describe pigs in management literature. This script flagged a total of 815 taxa for manual review. We did not include threats associated with domesticated pigs, but domestic pigs described as “free-ranging” were treated as wild. Similarly, some species were not threatened by pigs directly, but instead by human hunting practices catalyzed by the presence of pigs. These threats were noted but not included in the analysis.

The “Major Threats” section was then manually read, and cross referenced by two reviewers. False positives were flagged and removed from the subset of taxa identified as threatened by pigs for a final set of 672 taxa. Using the definition of “threat” provided by the IUCN Red List, all reference to threats from wild pigs hereafter can be defined as processes (e.g. predation, disturbance) that directly have “impacted, are impacting, or may impact the taxon being assessed”^5^. To ascertain the threat level from wild pigs to these taxa we used a similar approach to previous studies and categorized threat level as “major”, “minor”, or “potential” based on information in the “Major Threats” section text and the taxa’s current threat status^4,7,8^. We chose to include “potential” instead of “mixed” like many other studies due to uncertainty surrounding some of the threat text associated with the threatened taxa. Threats from wild pigs were sometimes inferred by the author of the listing based on overlapping distribution of the threatened species with wild pigs, but evidence of direct impact was sometimes missing. In these cases, threats from wild pigs were categorized as “potential”. When threats were associated with extinct or critically endangered taxa, text alluding to any threat from wild pigs resulted in a categorization as “major” unless sufficient detail was included to categorize the threat otherwise. Threats to least concern taxa were considered by default to be minor as were secondary threats to near-threatened taxa unless otherwise specified. For each taxon threatened by pigs, we categorized threat as one or more of the following categories: “predation”, “disturbance”, “disease risk”, “competition”, and “hybridization”. Unless otherwise specified, consumption of plants by wild pigs was considered both “predation” and “disturbance”, as it impacts the vegetation structure in addition to directly impacting the plants. Similarly, digging up of nests of herpetofauna (reptiles and amphibians) and ground nesting birds was counted as both “predation” and “disturbance”, as it results in disturbance of the nest site, in addition to direct mortality to herpetofauna and birds.

**Figure 1:**
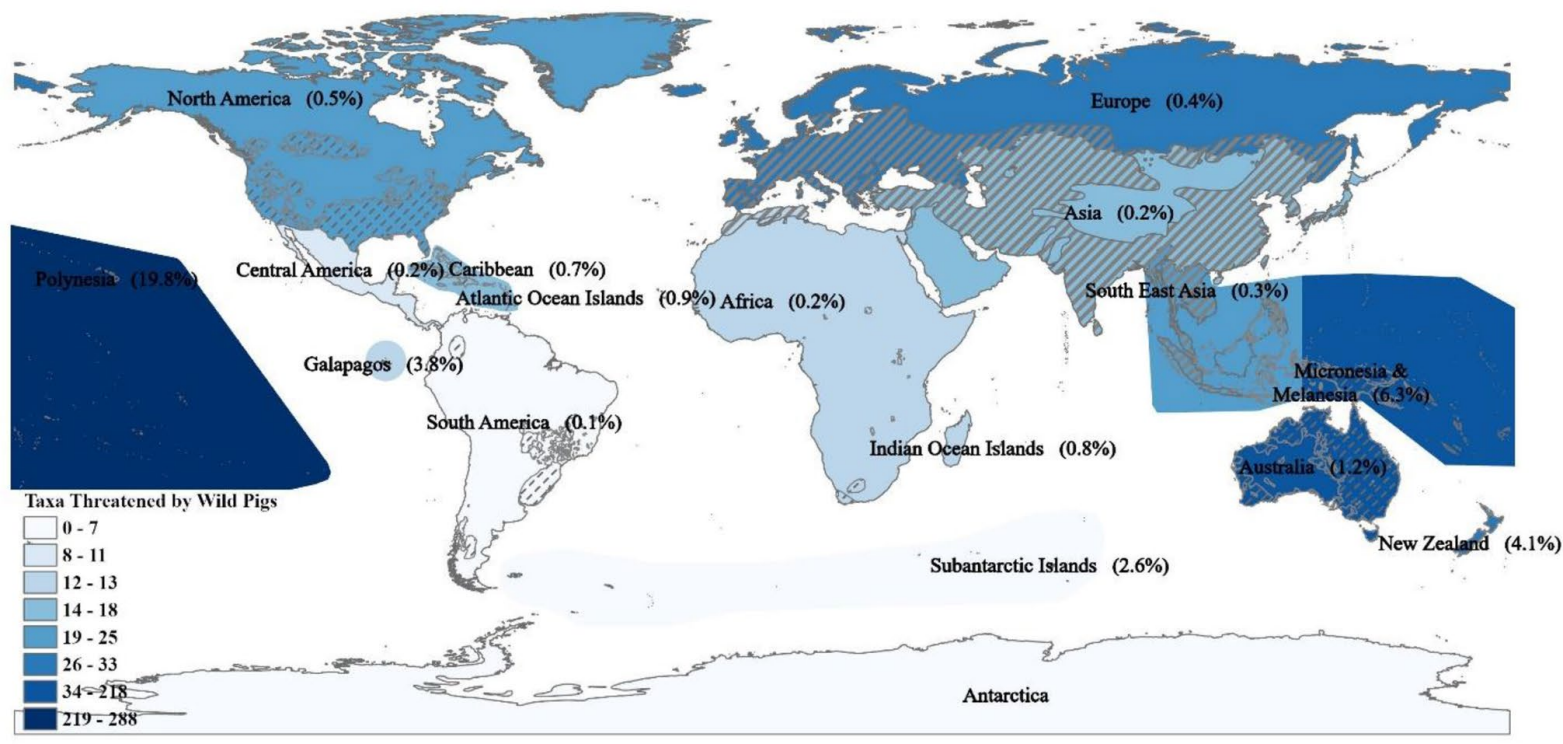
Number of taxa described as threatened by wild pigs on the IUCN Red List of Threatened Species for each of the 17 regions. Percentage of taxa threatened by wild pigs in relation to all taxa present in each region is included in parentheses. The non-native ranges provided by Lewis et al. 2017 (dotted hatched areas) and native ranges provided by the IUCN Red List (solid hatched areas) are shown. This map was created using ArcGIS Pro version 2.5.0 (https://www.esri.com/en-us/arcgis/products/arcgis-pro/overview).

Range information for each taxon obtained from the IUCN Red List were categorized into 17 different regions (Fig. 1). These regions were additionally classified as either island or continental based on their geographic location, to allow for a comparative threat analysis. Since IUCN Red List range data are classified by country, many endemic species occurring on islands were cross listed as occurring on both the continent-based country which governed the island and the island on which they were present. These cross-listings would have overinflated the threats occurring in continental regions, in cases where species occur on an island but not on the continental portion of the country. Using the built-in filter functions in Microsoft Excel and more detailed range information from the “Range Description” text from the IUCN Red List, each of the cross-listed taxa were manually filtered by reading the range information for each taxon. Through this process, records of endemic species outside of their actual range were removed.

To identify threats to island endemics and continental taxa, each of the 17 different regions were categorized as either an island or continental system. The continental subset included all taxa on continental systems that occurred within either the non-native or native ranges of wild pigs. The island subset was filtered to only include species endemic to each of the island regions (e.g. Polynesia). For this analysis, species that occur on both islands and continental regions were only included in the continental threat assessment and removed from the island subset. The proportion of threatened taxa for each region (n = 17 regions) were then calculated by dividing the total number of taxa present in the region by the number of taxa threatened by pigs in the region. The mean proportion of threatened taxa and standard deviations were then calculated for island endemics (n = 8 regions) and continental taxa (n = 9 regions) using summary statistics in the dplyr package version 0.8.3 in R version 3.6.0^35,36^. To quantify the threats to taxa within the non-native and native ranges of wilds pigs, the proportion of total taxa to threatened taxa was calculated for each range. Taxa threatened by wild pigs with ranges overlapping both the native and non-native ranges of wild pigs were considered threatened in both.

## Results

### Global threat from wild pigs to biodiversity

**Figure 2:**
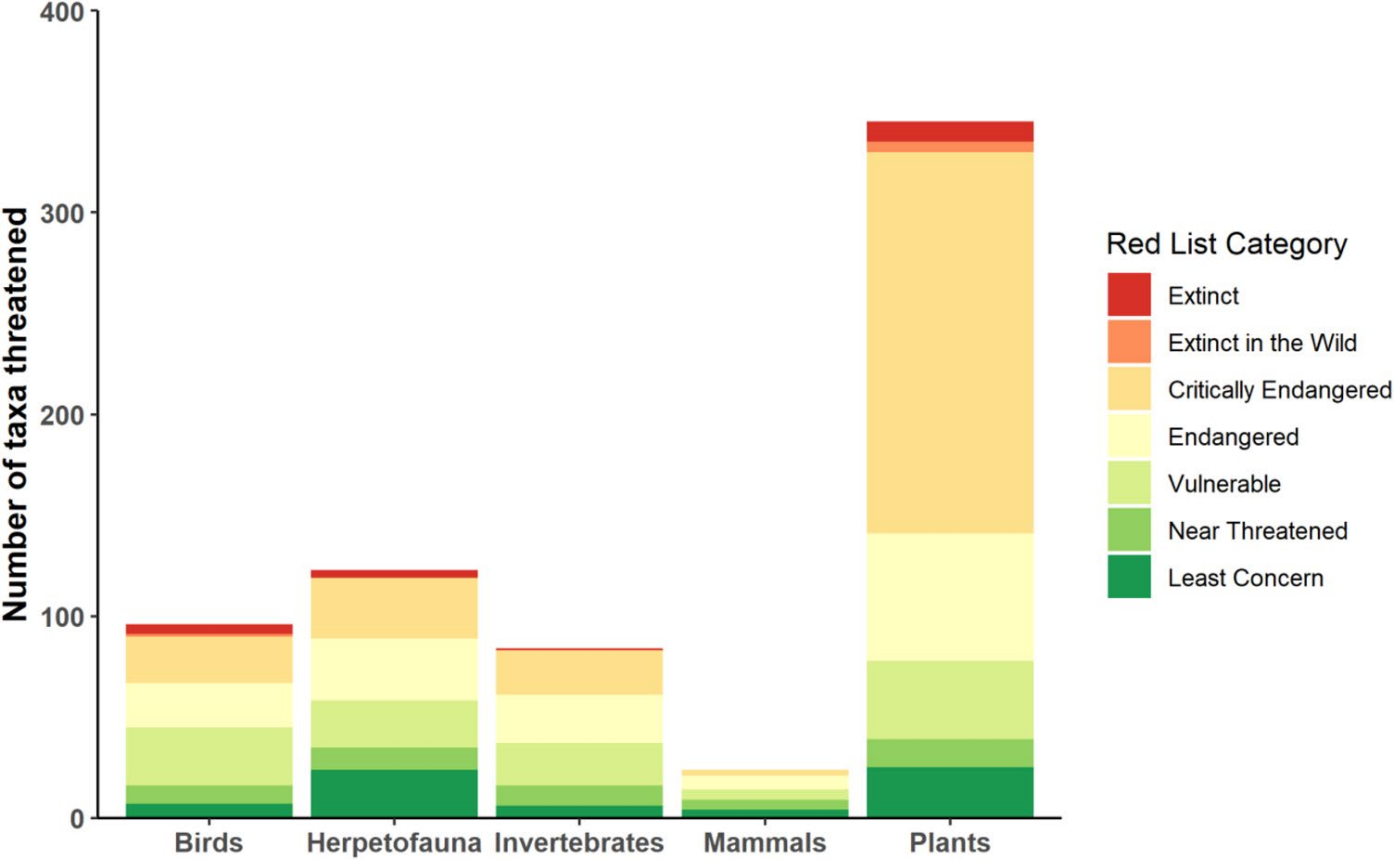
Quantity of taxa categorized by their IUCN Red List Category (fill colors) including all majorly, minorly, or potentially threatened taxa by wild pigs on the IUCN Red List. Taxa shown include all threatened taxa within the native or non-native ranges of wild pigs.

**Figure 3:**
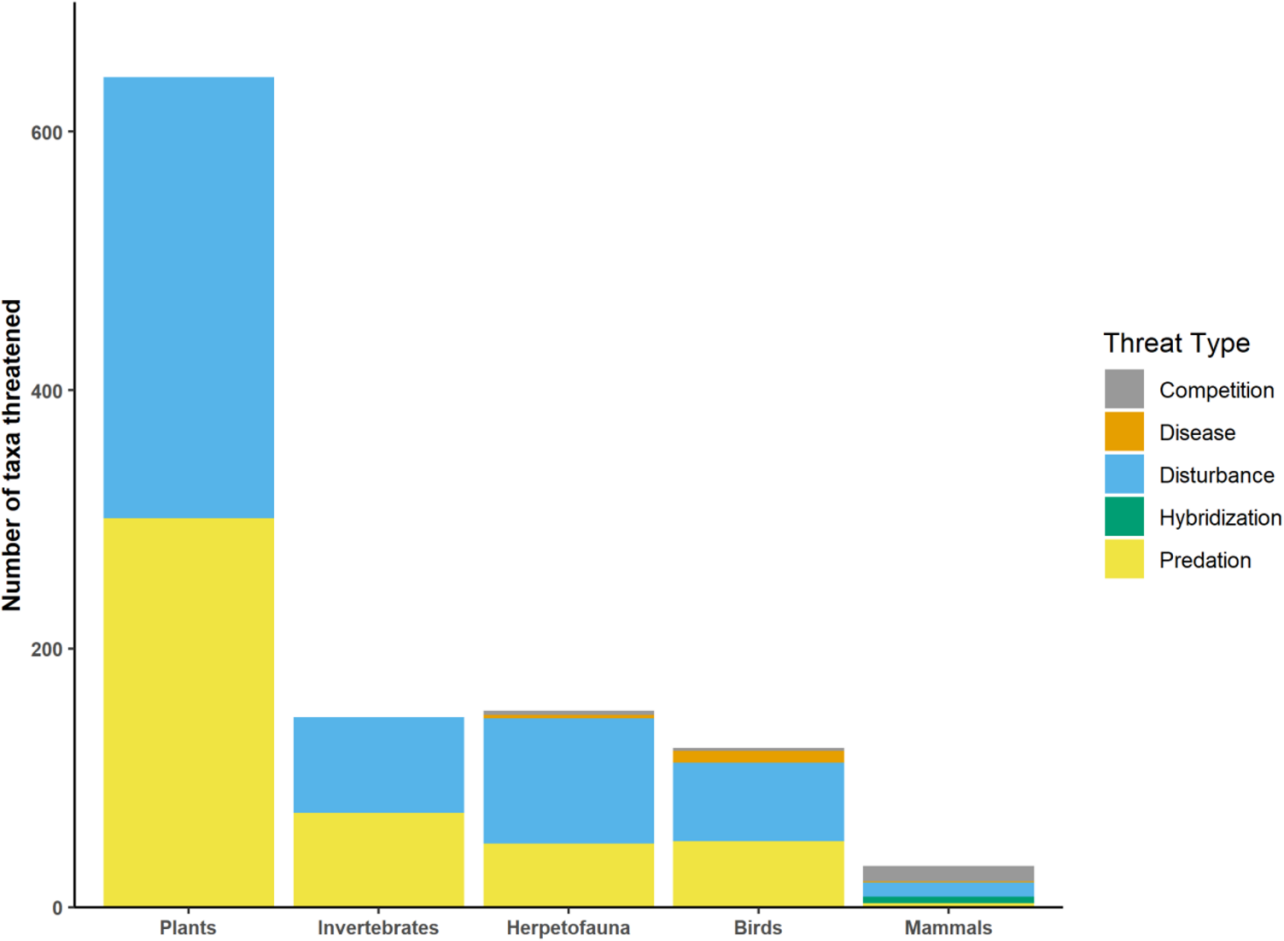
Quantity of all taxa on the IUCN Red List that are (extant) or have been (extinct), threatened by different wild pig threat types (e.g. Disturbance). Fill colors are the number of taxa for each taxonomic group.

Wild pigs were documented as a threat to 672 taxa from 54 different countries. Of these, 267 taxa were classified as critically endangered, 147 taxa were endangered, and 14 extinct taxa had classified pigs as a major contributing factor to their decline (Fig. 2 and Supplementary Table 1). Disturbance of habitat threatened 584 taxa making it the most frequently cited threat type, followed by 477 taxa threatened by predation. All other threat types affected less than 20 taxa (Fig. 3). Of the 672 taxa threatened by pigs, 345 were plants (59 families), 123 herpetofauna (25 families), 96 birds (38 families), 84 invertebrates (22 families), and 24 mammals (11 families) (Fig. 2). In total, 59% of threatened taxa faced major threats, 21% faced minor threats, and 20% were potentially threatened by wild pigs. Interestingly, a quarter (25%) of all taxa threatened by wild pigs were distributed amongst three plant families and one reptile family; the bellflower family (*Campanulaceae*), the palm family (*Arecaceae*), the daisy family (*Asteraceae*), and the skink family (*Scincidae*) (Supplementary Table 2). Bellflowers were the taxonomic family most threatened by wild pigs with 48 taxa classified as critically endangered or endangered and six either extinct or extinct in the wild (Supplementary Table 2).

### Threats to continental or island endemic taxa

**Figure 4:**
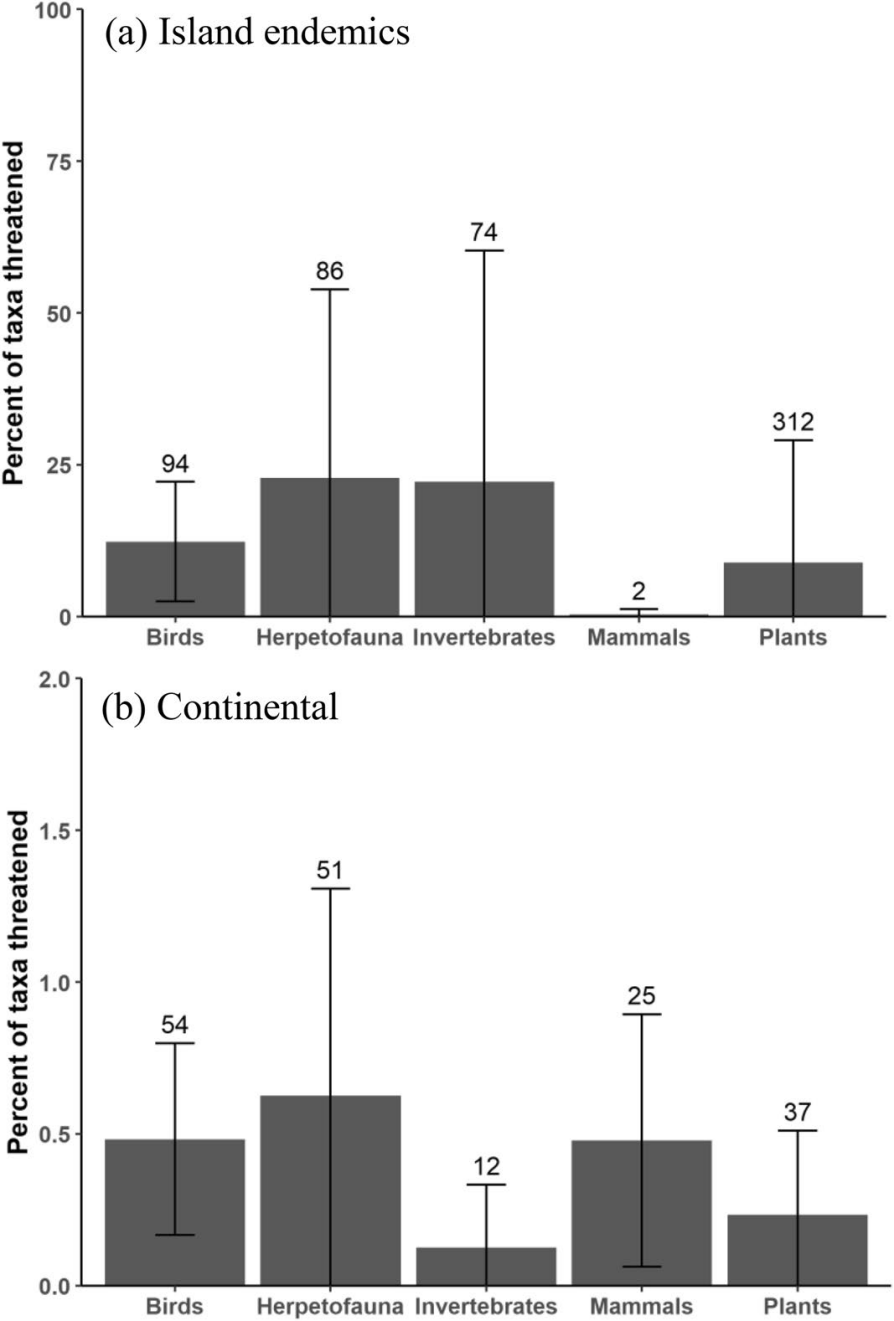
Mean percentages of all extant or extinct taxa identified as being threatened by wild pigs on the IUCN Red List across all (**a**) island endemic taxa (n = 8 regions) and (**b**) continental taxa (n = 9 regions). Numbers above errors bars are the total number of (**a**) island endemic taxa and (**b**) continental taxa threatened by wild pigs for each taxonomic group. Bars are ± 1 SD. Note: scales for (**a**) endemic taxa are 0–100% and (**b**) continental taxa are 0–2%. See “Methods” for details.

Wild pigs in island regions had stronger negative impacts on island endemic biodiversity when compared to continental regions (Fig. 4). In proportion to the total number of assessed continental and island endemic taxa, herpetofauna (22.8 ± 31.1%) invertebrates (22.2 ± 38.1%), and birds (12.3 ± 9.8%) were the most threatened island endemic taxa while herpetofauna (0.6 ± 0.7%), mammals (0.5 ± 0.4%), and birds (0.5 ± 0.3%) were the most threatened continental taxa (Fig. 4). Collectively, the Micronesian/Melanesian and Polynesian regions had the highest severity of endemic taxa threatened by wild pigs out of all regions. For the Micronesian/Melanesian region, 46% of all invertebrates (64 taxa), 25% of herpetofauna (65 taxa), 7% of plants (58 taxa), and 4% of all birds (25 taxa) were listed as threatened by wild pigs. The Polynesian region had 58% of plants (248 taxa) threatened, 25% of herpetofauna (3 taxa), and 19% of birds (29 taxa) threatened by wild pigs. Notably, 81% (9 taxa) of all assessed endemic herpetofauna in the Galapagos region were threatened by wild pigs with over half of them (5 taxa) belonging to the *Testudinidae* (tortoise) family. Additionally, 33% (3 taxa) and 12% (19 taxa) of endemic birds were threatened by wild pigs in the Galapagos and New Zealand respectively. For continental regions, Australia faced the highest threat rates with 1.1% of all birds (8 taxa), 0.6% of invertebrates (4 taxa), 1.1% of mammals (4 taxa), 0.7% of plants (8 taxa), and 2.1% of all herpetofauna (27 taxa). In the Australian region, the families most threatened by wild pigs were Australian ground frogs (*Myobatrachidae*) with 10 taxa, tree frogs (*Hylidae*) with 6 taxa, and orchids (*Orchidaceae*) with 4 taxa (Supplementary Table 3). North America and Europe were the next most threatened continental regions. More taxa were threatened in Europe than in North America but both regions experienced similar threat rates (proportion of taxa threatened by wild pigs to the total number of taxa). In Europe, 0.7% of birds (7 taxa), 0.2% of invertebrates (6 taxa), 0.5% of plants (16 taxa), and 0.9% of herpetofauna (4 taxa) were threatened by wild pigs. No mammals in Europe were listed as being threatened by wild pigs. In North America, 0.6% of all birds (6 taxa), 0.1% of invertebrates (1 taxa), 0.5% of mammals (2 taxa), 0.4% of plants (7 taxa), and 0.9% of herpetofauna (5 taxa) were under threat from pigs (Fig. 4). The most threatened families in Europe were land snails (*Helicidae*) and orchids (*Orchidaceae)*. Orchids were also the most threatened taxonomic family in North America and beeches and oaks (*Fagaceae*) were the second most threatened family by wild pigs.

### Threats throughout the native and non-native range of wild pigs

**Figure 5:**
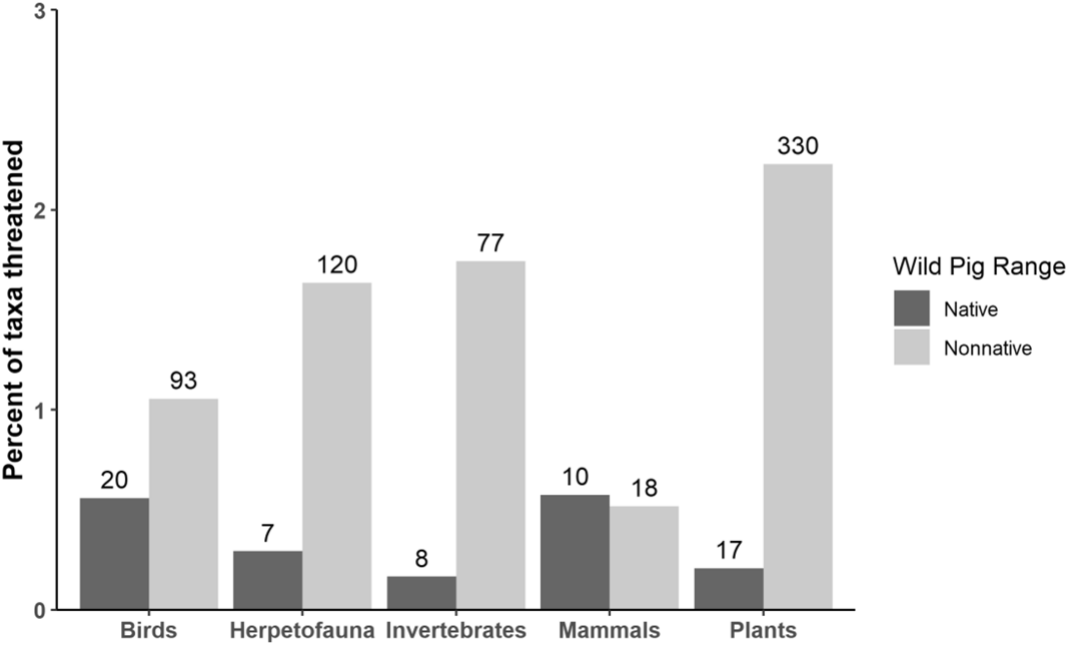
Percent of all taxa (extant or extinct) described as being threatened (major, minor, or potential) by wild pigs on the IUCN Red List within their native (dark gray bars) and non-native (light gray bars) range. Numbers above bars are the total number of taxa threatened by wild pigs for each category.

Threats to taxa within the native and non-native range of wild pigs were also markedly different. A total of 62 taxa and 638 taxa were listed as threatened by wild pigs within their native and non-native ranges respectively (Fig. 5). There were 28 unique taxa that were threatened in both the native and non-native ranges of wild pigs. In the native range of wild pigs, 0.6% of all birds (20 taxa), 0.6% of mammals (10 taxa), 0.3% of herpetofauna (7 taxa), 0.2% of plants (17 taxa), and 0.2% of invertebrates (8 taxa) were listed as threatened by wild pigs (Fig. 5). The taxonomic families with the most individuals threatened by wild pigs within their native range were; seabirds (*Procellariidae*) with 5 taxa, land snails (*Helicidae*) with 4 taxa, orchids (*Orchidaceae*) with 3 taxa, and pigs (*Suidae*) with 3 taxa. In the non-native range of wild pigs, 2.3% of all plants (330 taxa), 1.7% of invertebrates (77 taxa), 1.7% of herpetofauna (120 taxa), 1.1% of birds (93 taxa), and 0.5% of mammals (18 taxa) were listed as being threatened by wild pigs (Fig. 5). The most threatened families within the non-native range of wild pigs were bellflowers (*Campanulaceae*) with 57 taxa, skinks (*Scincidae*) with 46 taxa, palms (*Arecaceae*) with 38 taxa, and daisies (*Asteraceae*) with 26 taxa.

## Discussion

Our study is the most comprehensive analysis to date that quantifies the threat to global biodiversity from wild pigs to both continental taxa and island endemics across their native and non-native range, considering a multitude of threatening processes and all terrestrial taxa with sufficient assessment information on the IUCN Red List. Our assessment indicates that wild pigs are non-discriminant generalists, threatening 672 taxa globally, and have had a major contribution to the extinctions of 14 taxa. Over half (*n* = 414 taxa) of all taxa threatened by pigs are listed as either critically endangered or endangered and are of the greatest conservation concern. The disproportionate threat to at-risk species may be an expected result as threat assessments are generally more complete for species of concern. However, the quantity of at-risk species threatened by wild pigs represents a serious threat to biodiversity that has been indicated throughout the literature but had not been previously quantified. The estimates from our assessment are likely conservative due to the exclusion of data deficient species in our analysis and known biases associated with threat reporting and species assessments, such as the disproportionate lack of assessments for plants, invertebrates, amphibians and reptiles^34,37,38^. Furthermore, wild pigs have highly destructive behaviors that cause cascading trophic effects which broadly impact ecosystems, yet these threats are not easily quantified and most likely are largely excluded from species assessments^13,25,39^.

Impacts from wild pigs to island species are particularly acute, especially in the Polynesian region. This result is overwhelmingly driven by taxa in the Hawaiian Islands, since 92% of all taxa threatened by pigs in this region occur on the Hawaiian Islands. This is likely due to data deficiencies in species assessments on other Polynesian islands^40^. As data deficient species and regions are typically of high conservation concern^34,41^, the threats to the broader Polynesian region as well as other data deficient regions are likely to be greater than identified in this study.

Proportionately, island endemic invertebrates and herpetofauna are the taxa most threatened by wild pigs. Herpetofauna are threatened by both direct predation and disturbance to nest sites. Herpetofauna endemic to the Galapagos and Micronesian/Melanesian region were found to suffer higher threat rates from wild pigs than elsewhere (81% and 25%, respectively). For island regions with insufficient species assessments, this is particularly concerning as herpetofauna present there may be more threatened by wild pigs than indicated by this assessment. More comprehensive species assessments and research attention to island herpetofauna is needed as they are one of the most data deficient taxonomic groups on the Red List^32,42,43^. Generally, plants had the highest number of taxa threatened by pigs, with this result driven by species endemic to Polynesia (*n* = 248 taxa) or Micronesia/Melanesia (*n* = 58 taxa). Island native and endemic plants are most likely threatened in these regions due to the absence of analogous terrestrial mammalian omnivores throughout their evolutionary history and a propensity for island systems to host considerably higher densities of wild pigs^33,44^. As a result, many island plant species are exposed to an elevated level of threat from pigs and simultaneously lack the evolutionary traits and behaviors that could protect them against omnivorous ungulates^45,46^. The same is likely true for island herpetofauna, leaving them naïve to threats from wild pigs^47–49^.

In comparison, the highest proportionately threatened continental taxa by wild pigs were herpetofauna, mammals, and birds (Fig. 5). An overwhelming majority of the continental herpetofauna threatened by wild pigs occur in Australia. Australian ground frogs (*Myobatrchidae*) and tree frogs (*Hylidae*) were the most threatened taxonomic families throughout Australia, and Australian ground frogs had the highest number of individuals impacted out of any continental taxonomic family. We found few studies directly addressing the impacts wild pigs have on these vulnerable populations and several of the listings for these taxa explicitly mention the lack of research into the extent of these impacts^50^. The lack of research into the threats herpetofauna face across both continental and island ecosystems and the fact that herpetofauna rank as one of the most impacted taxonomic groups by wild pigs further elucidates the dire need to further understand the mechanisms of these species’ decline^37^. We found that the continental regions facing the next highest threat rates from wild pigs behind Australia were Europe and North America. Wild pigs are native to Eurasia and thus are not considered an invasive species throughout Europe yet are considered a pest in many parts throughout their native range due to unmanaged populations and a current lack of historical predators^13^. Interestingly, we found that wild pigs impact similar quantities of taxa and taxonomic groups in both North America and Europe despite the fact that pigs are native to Europe and considered invasive to North America.

Our analysis suggests nearly five times more taxa are threatened by wild pigs than an IUCN meta-analysis conducted by Doherty et al. (2016). However, the analysis conducted by Doherty et al. 2016 was primarily concerned with the impacts of invasive predators, and so excluded taxa that were predominantly threatened by non-predatory behaviors. We believe that incorporating both plant taxa and amphibians (excluded from Doherty et al. 2016), which rank among the highest threatened taxa throughout their native and non-native range, was crucial in identifying the full extent of threats to biodiversity from wild pigs due to their broad ecosystem-level impacts and omnivorous diet. Our keyword search also included a wider range of search terms than previous studies, potentially due to preliminary consultation with IUCN indicating a wide range of terms used to describe *Sus scrofa*. Even so, the list of taxa impacted by pigs is likely an underestimate, as plants, invertebrates and herpetofauna are often data deficient, or have not been assessed by the IUCN Red List of Threatened Species. Additionally, pigs have been present in many island ecosystems for long periods of time; thus, historical declines and extinctions caused by the introduction of pigs may be poorly documented or absent.

Threats from wild pigs as indicated by this assessment place them among the most problematic invasive predators that have undergone similar analyses^4,6,7^. Many of these assessments exclude threats to terrestrial invertebrates, amphibians, and plants as well as any threats to species of least concern or near threatened status. For direct comparison, if these criteria are excluded from our dataset, wild pigs still are identified as threatening 167 taxa globally (72 reptiles, 80 birds, 15 mammals) ranking them among some of the world’s top invasive predators such as domestic dogs (Supplementary Table 6)^6^. Also, threats to island species from wild pigs rank closely to feral cats in terms of the number of species impacted, despite a well-deserved reputation regarding cats as the most detrimental invasive predator to island ecosystems^31^. Medina et al. (2011) identified 175 taxa threatened by feral cats on islands, while our assessment indicates wild pigs threaten at least 131 taxa (63 reptiles, 65 birds, 3 mammals) using the same criteria (Supplementary Table 5). Given the role of wild pigs as both a top predator and destructive herbivore, their additional threats to plant and invertebrate taxa make them a serious cause for concern and indicate major ecosystem level impacts^51^. Furthermore, wild pigs not only threaten a comparable number of taxa as other invasive predators, they impact taxonomic groups that are often minimally threatened by other invasive mammalian species, such as herpetofauna and plants^52^.

The implications of this study highlight the importance of ongoing efforts to increase the accuracy of the IUCN Red List of Threatened Species and represent an extensive body of work compiled by numerous researchers across the globe. Global assessments like those described in this study would not be possible without the work of numerous IUCN assessors who contribute to the IUCN Red List. However, this assessment also highlights deficiencies within this extensive dataset and the implications they may have for assessing threats to biodiversity. Other approaches to threat assessments of wild pigs have been conducted at more regional scales and may provide additional information regarding the potential for wild pig impacts that might not currently be described within the IUCN Red List. For example, McClure et al. were able to describe the potential threat from wild pigs to species of concern at present day and future distribution scenarios within the contiguous United States^11^. In contrast to our assessment, McClure et al. used overlapping range information of wild pigs and species of concern to indicate potential risk to imperiled species. They found similar patterns in the most threatened taxonomic groups within the United States; however, they describe considerably more species at-risk from wild pigs than indicated by this assessment. The approaches used by McClure et al. describe potential threats based on overlapping ranges while the approaches used in this assessment describe documented threats to taxa assessed on the IUCN Red List. However, despite the differences in approach, the contrast between documented threats to taxa on the IUCN Red List and the potential for threat described by McClure et al. might be indicative of deficiencies in threat assessments for taxa throughout the region. This disparity may provide valuable information to address current deficiencies in threat assessments in a more targeted manner.

Given the extensive threats documented by this assessment, there are multiple ways to effectively manage wild pigs on both island and continental systems throughout their native and non-native ranges. In many cases, wild pigs are an abatable threat, with available management actions like exclusion fencing, baiting, trapping, and eradication on islands and from protected areas^48^. Island regions, which are most threatened by the presence of wild pigs, have benefited from successful eradication campaigns; the subsequent recovery of native species are indicative of major ecosystem level impacts associated with their presence^53^. Eradication efforts have even been successful for larger islands (> 100 km^2^) where threatened endemic species are beginning to recover^15,54,55^. Although quantitative information on native species recovery following island-wide eradication is uncommon^32^, Donlan et al. found considerable increases in the density of the endemic Galapagos rail (*Laterallus spilonotus*) after goat and pig eradication^56^. Where eradication is not feasible, such as continental systems or larger islands, other adaptive management approaches in the form of targeted control efforts^57,58^ and protected refuges using exclusion fencing have helped alleviate pig pressures on vulnerable taxa^17,59^. Furthermore, recent efforts to predict the distribution of wild pigs at various spatial and temporal scales show promise in prioritizing control efforts to areas most in need^33,60–62^. However, given the available management tools, the amount of conservation effort dedicated toward wild pig management on islands in particular is disproportionate to the threats species face, as evidenced by our assessment and previous studies^32^. Few islands include comprehensive pig management for the purposes of conservation; furthermore, wild pigs have only been eradicated from 69 islands^63^. In comparison, cats (*Felis* catus) have successfully been eradicated from 148 islands and feral goats (*Capra* hircus) have successfully been eradicated from 195 islands^63^. Our assessment suggests that pig control efforts on island ecosystems would benefit the greatest number of species, particularly throughout Polynesia, Micronesia, and Melanesia. Additionally, special concern should be placed on islands with a diverse presence of herpetofauna, invertebrates, or endemic plant species due to their vulnerability. Finally, more research attention should be focused on the assessment of herpetofauna as they are often data deficient and threats to these taxa are likely far greater than currently represented in species assessments.

## Supplementary Information

Supplementary Tables.
