# Supplementary material for: The global impact of wild pigs (Sus scrofa) on terrestrial biodiversity
Source: Sci Rep. 2021 Jun 24;11:13256. doi: 10.1038/s41598-021-92691-1 (PMC8225776; doi:10.1038/s41598-021-92691-1)
Supplement: Supplementary file 1 — Supplementary Tables. [file 41598_2021_92691_MOESM1_ESM.docx]

**Supplementary information to:**

**The global impact of wild pigs (*Sus scrofa*) on terrestrial biodiversity**

Derek R. Risch, drisch@hawaii.edu^1^

Jeremy Ringma, jeremy.ringma@gmail.com^1,2^

Melissa R. Price, pricemel@hawaii.edu^1^

^1^University of Hawaiʻi at Mānoa, 1910 East-West Rd. HI 96822, USA

^2^Royal Melbourne Institute of Technology University, GPO Box 2476V, Melbourne 3001, AUS

Corresponding author: drisch@hawaii.edu

**Table 1.** Species with wild pigs (*Sus scrofa)* classified as a major contributing factor to their extinction.

| Species affected | Taxonomic group | Common name | Subregion (island) |
| --- | --- | --- | --- |
| *Melicope nealae* | Plants | - | Polynesia (Hawaiʻi) |
| *Cyperus rockii* | Plants | Kaua‘i flatsedge | Polynesia (Hawaiʻi) |
| *Cyanea sessilifolia* | Plants | - | Polynesia (Hawaiʻi) |
| *Gallinula nesiotis* | Birds | Tristan moorhen | Atlantic Island (Saint Helena) |
| *Coenocorypha iredalei* | Birds | South Island snipe | New Zealand |
| *Moho braccatus* | Birds | Kauaʻi ʻōʻō | Polynesia (Hawaiʻi) |
| *Argyroxiphium virescens* | Plants | Hawaiian Greensword | Polynesia (Hawaiʻi) |
| *Hibiscadelphus woodii* | Plants | - | Polynesia (Hawaiʻi) |
| *Delissea niihauensis* | Plants | - | Polynesia (Hawaiʻi) |
| *Melicope macropus* | Plants | - | Polynesia (Hawaiʻi) |
| *Cyanea superba* ssp. *regina* | Plants | Mt. Kaala cyanea | Polynesia (Hawaiʻi) |
| *Taudactylus diurnus* | Herpetofauna | Mount Glorious day frog | Australia |
| *Chilonopsis nonpareil* | Invertebrates | - | Atlantic Island (Saint Helena) |
| *Tetramolopium consanguineum* subsp. *consanguineum* | Plants | - | Polynesia (Hawaiʻi) |

**Table 2.** Global top ten (out of 155 total families) most impacted taxonomic families by wild pigs (*Sus scrofa*).

| Family affected | Taxonomic group | Common name | Extinct | Extinct in the wild | Critically Endangered | Endangered | Vulnerable | Near Threatened | Least Concern | Total affected |
| --- | --- | --- | --- | --- | --- | --- | --- | --- | --- | --- |
| *Campanulaceae* | Plants | Bellflower family | 3 | 3 | 39 | 9 | 3 | 0 | 0 | 57 |
| *Scincidae* | Herpetofauna | Skink family | 1 | 0 | 10 | 14 | 10 | 3 | 9 | 47 |
| *Arecaceae* | Plants | Palm family | 0 | 0 | 9 | 12 | 4 | 3 | 10 | 38 |
| *Asteraceae* | Plants | Daisy family | 2 | 0 | 18 | 4 | 4 | 0 | 0 | 28 |
| *Diplodactylidae* | Herpetofauna | Gecko family | 0 | 0 | 7 | 6 | 3 | 6 | 2 | 24 |
| *Lamiaceae* | Plants | Mint family | 0 | 0 | 17 | 0 | 0 | 0 | 1 | 18 |
| *Charopidae* | Inverts | Land snail family | 0 | 0 | 6 | 3 | 7 | 0 | 0 | 16 |
| *Procellariidae* | Birds | Seabird family | 0 | 0 | 5 | 3 | 7 | 1 | 0 | 16 |
| *Caryophyllaceae* | Plants | Pink family | 0 | 0 | 14 | 1 | 0 | 0 | 0 | 15 |
| *Pittosporaceae* | Plants | Cheese- wood family | 0 | 0 | 0 | 1 | 7 | 5 | 1 | 14 |

| Family affected | Taxonomic group | Common family name | Extinct | Extinct in the wild | Critically Endangered | Endangered | Vulnerable | Near Threatened | Least Concern | Total affected |
| --- | --- | --- | --- | --- | --- | --- | --- | --- | --- | --- |
| *Myobatrachidae* | Herpetofauna | Australian ground frog | 2 | 0 | 4 | 2 | 2 | 0 | 0 | 10 |
| *Orchidaceae* | Plants | Orchid family | 0 | 0 | 0 | 0 | 3 | 2 | 3 | 8 |
| *Hylidae* | Herpetofauna | Tree frog family | 0 | 0 | 2 | 3 | 0 | 1 | 0 | 6 |
| *Suidae* | Mammals | Pig family | 0 | 0 | 2 | 3 | 0 | 1 | 0 | 6 |
| *Cervidae* | Mammals | Deer family | 0 | 0 | 1 | 0 | 1 | 2 | 0 | 4 |
| *Helicidae* | Invertebrates | Land snail family | 0 | 0 | 1 | 2 | 1 | 0 | 0 | 4 |
| *Scincidae* | Herpetofauna | Skink family | 0 | 0 | 0 | 2 | 0 | 1 | 1 | 4 |
| *Elapidae* | Herpetofauna | Snake family | 0 | 0 | 0 | 0 | 0 | 0 | 3 | 3 |
| *Fabaceae* | Plants | Pea family | 0 | 0 | 1 | 1 | 1 | 0 | 0 | 3 |
| *Turnicidae* | Birds | Button-quail family | 0 | 0 | 0 | 1 | 0 | 1 | 1 | 3 |

**Table 3.** Continental top ten (out of 77 total families) most impacted taxonomic families by wild pigs (*Sus scrofa*).

**Table 4.** Island top ten (out of 98 total families) most impacted taxonomic families by wild pigs (*Sus scrofa*).

| Family affected | Taxonomic group | Common name | Extinct | Extinct in the wild | Critically Endangered | Endangered | Vulnerable | Near Threatened | Least Concern | Total affected |
| --- | --- | --- | --- | --- | --- | --- | --- | --- | --- | --- |
| *Campanulaceae* | Plants | Bellflower family | 3 | 3 | 39 | 9 | 3 | 0 | 0 | 57 |
| *Scincidae* | Herpetofauna | Skink family | 1 | 0 | 10 | 12 | 10 | 2 | 8 | 43 |
| *Arecaceae* | Plants | Palm family | 0 | 0 | 9 | 12 | 4 | 3 | 10 | 38 |
| *Asteraceae* | Plants | Daisy family | 2 | 0 | 16 | 4 | 4 | 0 | 0 | 26 |
| *Diplodactylidae* | Herpetofauna | Gecko family | 0 | 0 | 7 | 6 | 3 | 6 | 2 | 24 |
| *Lamiaceae* | Plants | Mint family | 0 | 0 | 17 | 0 | 0 | 0 | 0 | 17 |
| *Charopidae* | Invertebrates | Land snail family | 0 | 0 | 6 | 3 | 7 | 0 | 0 | 16 |
| *Procellariidae* | Birds | Seabird family | 0 | 0 | 5 | 3 | 7 | 1 | 0 | 16 |
| *Caryophyllaceae* | Plants | Pink family | 0 | 0 | 14 | 1 | 0 | 0 | 0 | 15 |
| *Pittosporaceae* | Plants | Cheese-wood family | 0 | 0 | 0 | 1 | 7 | 5 | 1 | 14 |

**Table 5.** Number of island species threatened by wild pigs (*Sus scrofa*) for the five most critical categories of the IUCN Red List : EX, extinct; EW, extinct in the wild; CR, critically endangered; EN, endangered; VU, vulnerable. This table was created for comparison with Medina et al. (2011) meta-analysis of feral cats (*Felis* catus) impacts on islands. Subspecies are noted in brackets.

| Group | EX | EW | CR | EN | VU | Total | Total taxa |
| --- | --- | --- | --- | --- | --- | --- | --- |
| Reptiles | 2 | 0 | 23 | 21 (1) | 14 (2) | 60 (3) | 63 |
| Birds | 5 | 1 | 21 | 15 | 23 | 65 | 65 |
| Mammals | 0 | 0 | 0 | 3 | 0 | 3 | 3 |
| Total | 7 | 1 | 44 | 39 (1) | 37 (2) | 128 (3) | 131 |

**Table 6.** Number of species globally threatened by wild pigs (*Sus scrofa*) for the five most critical categories of the IUCN Red List: EX, extinct; EW, extinct in the wild; CR, critically endangered; EN, endangered; VU, vulnerable. This table was created using the same filtering criteria as used by Doherty et al. (2016). Subspecies are noted in brackets.

| Group | EX | EW | CR | EN | VU | Total | Total taxa |
| --- | --- | --- | --- | --- | --- | --- | --- |
| Reptiles | 2 | 0 | 24 | 24 (1) | 19 (2) | 69 (3) | 72 |
| Birds | 5 | 1 | 23 | 22 | 29 | 80 | 80 |
| Mammals | 0 | 0 | 3 | 7 | 5 | 15 | 15 |
| Total | 7 | 1 | 50 | 53 (1) | 53 (2) | 164 (3) | 167 |
